# Cancer Metabolism and Its Historical & Molecular Foundations: An Overview

**DOI:** 10.3390/ddc5010017

**Published:** 2026-03-01

**Authors:** Rami A. Al-Horani

**Affiliations:** Division of Basic Pharmaceutical Sciences, College of Pharmacy, Xavier University of Louisiana, New Orleans, LA 70125, USA

**Keywords:** metabolic reprogramming, aerobic glycolysis, Warburg effect, TME, mitochondrial dynamics, epigenetic regulation, targeted therapy

## Abstract

Cancer metabolism is a cornerstone of tumor biology, characterized by profound alterations in cellular energy production and biosynthetic pathways that drive malignancy. The seminal discovery of the “Warburg effect”, the preference of cancer cells for aerobic glycolysis even under oxygen-rich conditions, provided the first major insight into this field. Historically, this observation was attributed to defective mitochondria, but modern research has revealed a far more complex picture of metabolic reprogramming that is actively driven by oncogenes, tumor suppressor genes, and the tumor microenvironment (TME). This review advances a unifying framework for understanding cancer metabolism as a dynamic ecosystem defined by three interconnected adaptations: metabolic plasticity, oncometabolite-driven epigenetic remodeling, and immune-metabolic crosstalk. These adaptations extend beyond glycolysis to encompass glutamine metabolism, lipid synthesis, amino acid utilization, and mitochondrial dynamics, all coordinated to fuel rapid proliferation, promote survival, and enable metastasis. By examining the drivers, consequences, and therapeutic barriers within this framework, we highlight emerging strategies for precision intervention. Although understanding the mechanistic basis of these pathways has unveiled new therapeutic avenues, clinical translation has been limited by metabolic redundancy, microenvironmental buffering, and patient heterogeneity. Strategies such as metabolic inhibitors, dietary interventions, and immuno-metabolic combinations offer promising prospects for disrupting tumor growth when guided by biomarker-driven patient selection and emerging technologies, including spatial metabolomics and AI-driven network modeling.

## 1. Introduction: Cancer and Its Metabolic Demand

Cancer is a heterogeneous group of diseases characterized by the uncontrolled proliferation of atypical cells that evade normal growth regulatory mechanisms. This progression often involves invasion of surrounding tissues and metastasis [1]. Unlike infectious diseases, cancer is primarily instigated by internal changes, such as mutations in proto-oncogenes, tumor suppressor genes, and apoptotic regulators, which directly or indirectly alter cell metabolism [2]. The hallmarks of cancer include sustaining proliferative signaling, evading growth suppressors, resisting cell death, enabling replicative immortality, inducing angiogenesis, and activating invasion and metastasis, with later additions of deregulating cellular metabolism and avoiding immune destruction [3].

A core trait of cancer cells is their ability to reprogram metabolic pathways to meet the high bioenergetic and biosynthetic demands of rapid proliferation [4]. This metabolic rewiring ensures a supply of ATP, macromolecules (nucleotides, lipids, proteins), and maintenance of redox balance, even under adverse conditions such as nutrient scarcity or hypoxia [5]. The initiation and progression of cancer are driven not only by genetic mutations but also by the interplay between these mutations and environmentally available nutrients, whereby accumulated metabolites can further promote tumorigenesis [6,7]. The tumor microenvironment (TME) plays a pivotal role, inducing adaptive mechanisms such as nutrient scavenging and shaping metabolic crosstalk between cancer cells, immune cells, and stromal cells [8,9].

**Figure 1.**
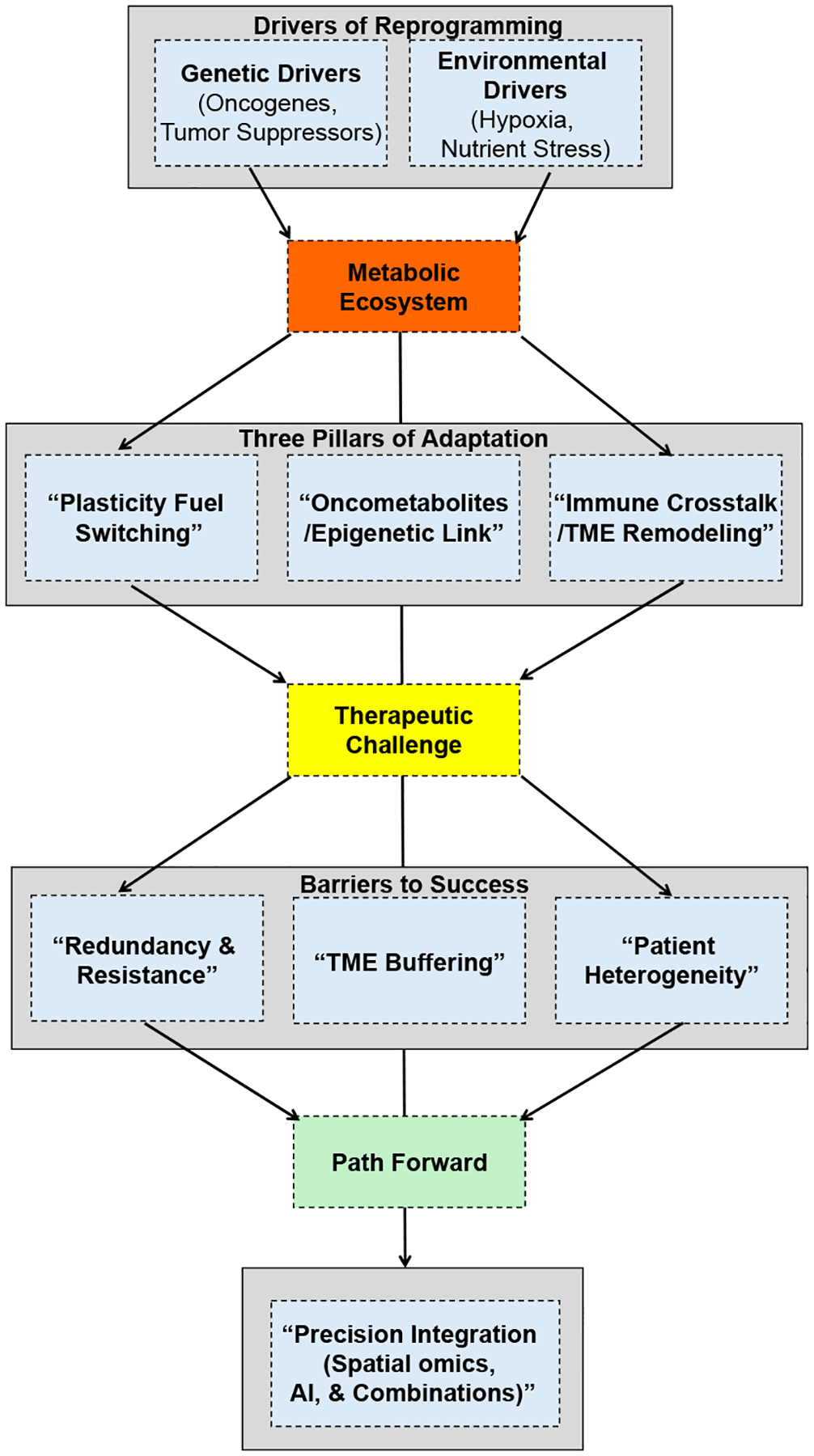
A unifying framework for cancer metabolism. This flow chart synthesizes the core concepts of the review. Cancer metabolism is driven by genetic and environmental factors, leading to a dynamic metabolic ecosystem characterized by three key adaptations: metabolic plasticity, oncometabolite production, and immune crosstalk. While these adaptations fuel tumor growth, they also generate therapeutic barriers, including pathway redundancy, microenvironmental buffering, and metabolic heterogeneity, that must be addressed through precision integration of emerging technologies.

To synthesize these complex concepts and provide a coherent roadmap for the reader, this review proposes a unifying framework that conceptualizes cancer metabolism as a dynamic ecosystem (Figure 1). This framework integrates the genetic and environmental drivers of metabolic change, the three core adaptive pillars of metabolic reprogramming, and the therapeutic barriers that must be overcome to achieve durable clinical responses. By organizing the extensive literature around this framework, we aim to move beyond a descriptive catalog of metabolic alterations toward a more integrated understanding that can inform future therapeutic strategies.

## 2. Historical Perspectives on Cancer Metabolism

The study of cancer metabolism has evolved through a cycle of discovery, skepticism, and revival, shaped by key historical observations. The history of cancer metabolism began in earnest in the 1920s with Otto Warburg, who observed that cancer tissue slices consume glucose avidly and ferment it to lactate, even in the presence of oxygen, a phenomenon termed aerobic glycolysis or the Warburg Effect [10–12]. Warburg postulated that this metabolic shift was due to irreversible damage to mitochondrial respiration, which he believed was the root cause of cancer [1,13]. While his central hypothesis of damaged mitochondria as the primary cause was later challenged, his core observation of altered glucose metabolism remains a fundamental pillar of cancer biology.

Beginning in the 1970s and continuing into the 1980s, cancer research underwent a major shift toward molecular oncology, driven by the discovery of cellular oncogenes and tumor-suppressor genes. Landmark studies during this period demonstrated that normal cellular genes such as RAS and MYC could be activated to drive malignant transformation, establishing the oncogene paradigm [14,15]. Likewise, the identification of TP53 (p53) in 1979 and its later recognition as a frequently mutated tumor-suppressor gene in human cancers further solidified the genetic basis of tumorigenesis [16]. These discoveries and others transformed cancer biology by revealing that mutations in specific genes are fundamental drivers of cancer development [17,18]. During this period, metabolic alterations were largely regarded as downstream consequences of oncogenic mutations rather than primary drivers of cancer. However, subsequent research demonstrated that oncogenes such as MYC and RAS directly reprogram cellular metabolism by transcriptionally regulating metabolic enzymes and transporters, thereby enhancing aerobic glycolysis, glutamine uptake, and glutaminolysis to support rapid tumor growth [19–21].

At the turn of the 21st century, enabled by rapid advances in metabolomics, genomics, and molecular biology, cancer metabolism experienced a major resurgence. It became increasingly evident that metabolic reprogramming is not merely a passive consequence of genetic alterations but a core hallmark of cancer, actively driven by oncogenic signaling pathways such as PI3K/AKT and HIF-1 to support anabolic growth, redox balance, and cell survival [22–26]. In other words, metabolic reprogramming refers to the coordinated alteration of cellular metabolic pathways that enables cancer cells to sustain proliferation, resist apoptosis, maintain redox balance, and adapt to environmental stress. Unlike passive metabolic changes, reprogramming is actively driven by oncogenes, tumor suppressor loss, epigenetic remodeling, and microenvironmental pressures, resulting in stable yet dynamic rewiring of central carbon metabolism. Today, the Warburg effect is understood not as a consequence of defective mitochondria but as a strategic metabolic adaptation. Aerobic glycolysis enables cancer cells to generate ATP rapidly while supplying essential metabolic intermediates to fuel biosynthetic pathways, such as the pentose phosphate pathway for nucleotide synthesis and the serine–glycine–one-carbon pathway for redox balance and methyl-group metabolism [27,28]. Modern research has expanded beyond glucose metabolism to highlight the central roles of glutamine addiction, de novo lipid synthesis, and the intricate metabolic crosstalk within the TME, where cancer cells compete with, and actively reprogram, immune cells to secure scarce nutrients [29,30]. This historical progression illustrates how revisiting foundational biochemical observations with contemporary molecular tools can reveal fundamental cancer vulnerabilities and inspire new therapeutic strategies.

## 3. Fundamentals of Cellular Metabolism and Its Rewiring in Cancer

**Figure 2.**
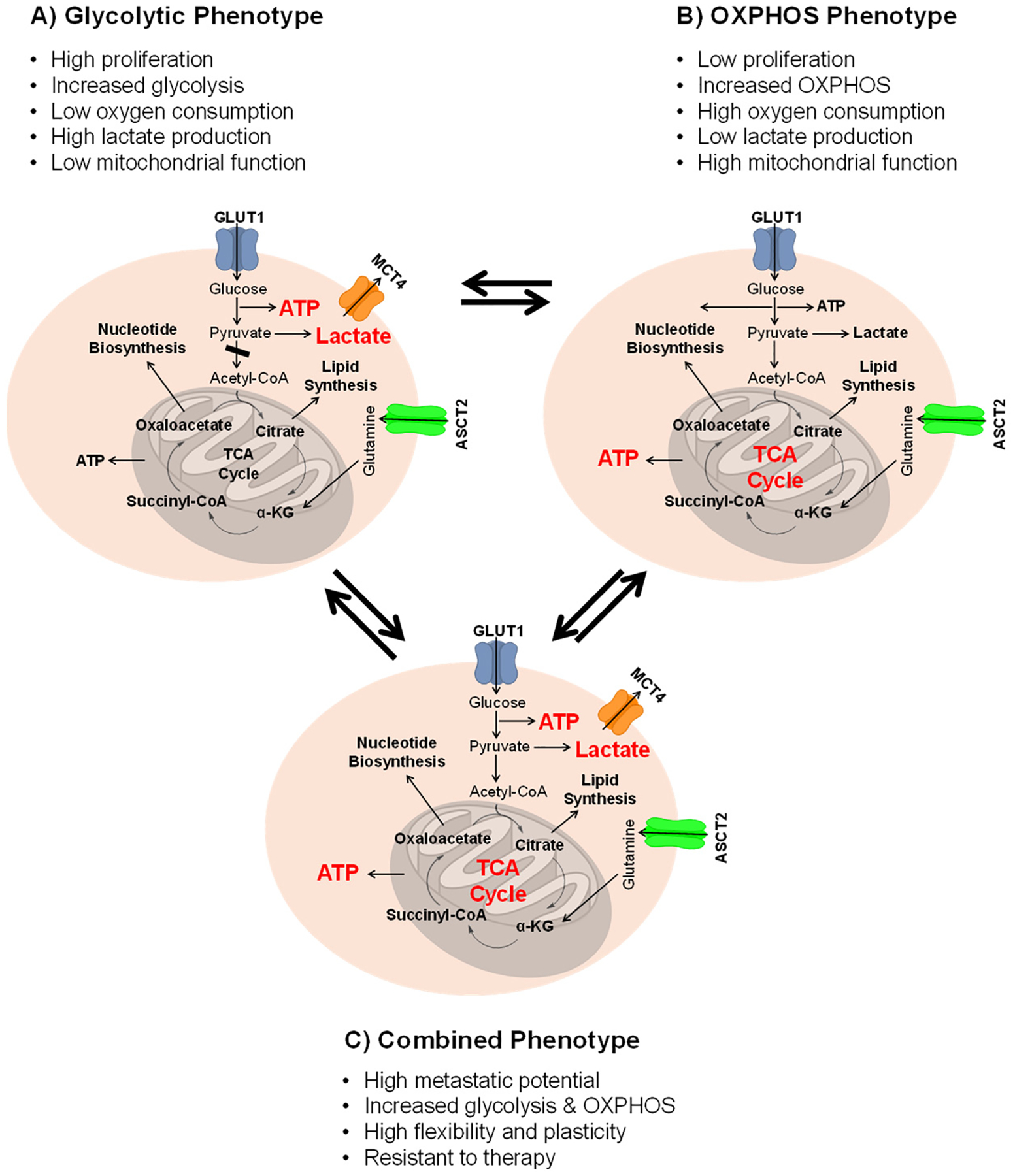
Metabolic states and their dynamic interconversion in cancer cells. Cancer cell metabolism is highly adaptable, with distinct metabolic phenotypes that can shift in response to varying cellular demands and environmental cues. (**A**) Glycolytic phenotype: High glucose uptake and lactate secretion. (**B**) Oxidative (OXPHOS) phenotype: Enhanced mitochondrial activity and oxygen consumption. (**C**) Hybrid phenotype: Simultaneous engagement of glycolysis and OXPHOS. Bidirectional arrows indicate the potential for dynamic transition between states in response to nutrient availability, oxygen tension, or therapeutic stress.

Normal cells maintain metabolic homeostasis by precisely regulating pathways such as glycolysis, the Krebs (tricarboxylic acid (TCA)) cycle, and oxidative phosphorylation (OXPHOS) in response to changing energy demands and nutrient availability (Figure 2) [31–33]. These processes are orchestrated by metabolic enzymes, energy sensors such as AMPK, and hormonal signals like insulin [34,35]. Cancer cells, however, profoundly disrupt this balance. They display pronounced metabolic plasticity, dynamically rewiring these core pathways to favor continuous biomass production and rapid proliferation, even within the nutrient-limited and hypoxic tumor microenvironment [25,36–38].

In the above figure, the glycolytic phenotype is characterized by a 10–20 fold increase in glucose uptake relative to normal tissue, prioritizing rapid ATP production and biosynthetic precursors. Conversely, the oxidative phenotype relies on mitochondrial NADH generation for efficient ATP synthesis. The hybrid phenotype represents a dynamic state where cells utilize both pathways, enabling adaptation to metabolic stress and therapeutic pressure. This ability to transition between states, metabolic plasticity, represents the first pillar of the adaptive ecosystem illustrated in Figure 1.

A central feature of the metabolic reprogramming is the shift in glucose metabolism. Cancer cells dramatically increase glucose uptake and glycolytic flux. This increase is so pronounced that it forms the basis for clinical FDG-PET imaging, where glycolytic tumors typically demonstrate a 10–20-fold increase in glucose uptake compared to adjacent normal tissue [39]. Glucose is preferentially converted to pyruvate by lactate dehydrogenase A (LDHA) rather than channeling it into the TCA cycle. This Warburg-like behavior provides a steady flow of carbon for biosynthesis and helps sustain redox balance [40–42]. Although early interpretations suggested mitochondrial dysfunction, mitochondria remain central to tumor metabolism. They support ATP production, enable anaplerotic replenishment of the TCA cycle—often through glutamine—regulate biosynthesis and redox signaling, and play key roles in apoptosis. Mutations in mitochondrial DNA and TCA-cycle enzymes such as Isocitrate Dehydrogenase (IDH) or Succinate Dehydrogenase (SDH) can produce oncometabolites that drive tumorigenesis [43–45].

Glutamine metabolism is another hallmark of cancer metabolic remodeling. Through glutaminolysis, glutamine supplies carbon and nitrogen to the TCA cycle, fuels nucleotide and amino acid synthesis, and supports redox homeostasis. Oncogenic drivers such as MYC amplify glutamine dependency by upregulating its transporters and catabolic enzymes [46–51]. Lipid metabolism is similarly reprogrammed: many tumors markedly enhance de novo lipogenesis—even when exogenous lipids are plentiful—to generate membranes, lipid-derived signaling molecules, and energy-dense lipid droplets. Enzymes such as fatty acid synthase (FASN) and transporters like CD36 (also known as fatty acid translocase or FAT) are frequently overexpressed to meet these demands [52–57].

Amino acid and one-carbon pathways also undergo substantial reprogramming. Increased serine, glycine, and one-carbon flux provide essential precursors for nucleotide biosynthesis and generate methyl donors such as S-adenosylmethionine required for DNA and histone methylation [58–60]. Furthermore, altered tryptophan metabolism through the kynurenine pathway fosters immune evasion by creating an immunosuppressive tumor microenvironment [61–63]. The Pentose phosphate pathway (PPP) adds another crucial layer to this metabolic reshaping. Its oxidative branch generates NADPH, which is essential for maintaining antioxidant defenses and supporting reductive biosynthesis, while its non-oxidative branch produces ribose-5-phosphate for nucleotide synthesis. By supplying both NADPH and ribose sugars, the PPP plays a critical role in managing oxidative stress and enabling the rapid growth characteristic of cancer cells [64–66].

Overall, these metabolic adaptations illustrate how cancer cells co-opt and remodel fundamental biochemical processes to sustain unchecked growth, survive hostile conditions, and evade immune surveillance. However, it is crucial to recognize that these phenotypes are not universal. For instance, many solid tumors, particularly those in hypoxic regions, exhibit a pronounced glycolytic (Warburg) phenotype. In contrast, some hematologic malignancies, like certain subtypes of acute myeloid leukemia (AML), and slow-growing or metastatic solid tumors, can be highly dependent on OXPHOS. Furthermore, within a single tumor, a gradient exists from glycolysis-dominant hypoxic cores to OXPHOS-dominant well-vascularized edges. This context-dependence dictates that therapeutic targeting must be tailored to the specific metabolic state of the tumor. For example, pancreatic ductal adenocarcinoma (PDAC), driven by KRAS, is addicted to macropinocytosis and glutamine metabolism, whereas clear cell renal cell carcinoma (ccRCC), driven by VHL loss, is characterized by profound lipid and glycogen accumulation. IDH-mutant gliomas represent a distinct class where the primary metabolic event is the production of an oncometabolite that drives epigenetic changes, rather than a shift in energy production per se. Recognizing this diversity is the first step toward precision metabolic oncology.

## 4. The Metabolic Characteristics of Nutrients in Cancer Cells

### 4.1. Glycolysis and TCA Cycle Alterations

Cancer cells undergo extensive metabolic reprogramming, characterized by enhanced glycolysis and disruption of the TCA cycle. The Warburg effect describes the preferential conversion of glucose to lactate even in the presence of oxygen, enabling rapid ATP production and generation of biosynthetic intermediates required for proliferation [67]. Lactate is not merely a metabolic byproduct but actively promotes tumor invasion, metastasis, angiogenesis, and immune modulation [68,69]. Through HIF-1α–dependent signaling, lactate induces vascular endothelial growth factor (VEGF) and arginase-1 (Arg1), promoting polarization of tumor-associated macrophages toward an M2, tumor-promoting phenotype [70–72]. In contrast, lactate has also been shown in certain contexts to enhance CD8^+^ T-cell effector function, underscoring its context-dependent immunological roles [73,74].

Despite the increased reliance of many tumors on aerobic glycolysis, mitochondrial metabolism remains indispensable for tumor growth. Beyond ATP generation, the TCA cycle functions as a biosynthetic hub, supplying precursors for nucleotide, amino acid, and lipid synthesis while maintaining redox homeostasis through NADH and NADPH production [75]. Consequently, genetic alterations affecting TCA cycle enzymes do not simply impair respiration but instead rewire mitochondrial metabolism in ways that generate oncometabolites and promote tumorigenesis. As summarized in Figure 1, these oncometabolites represent the second pillar of the metabolic ecosystem, directly linking altered metabolic flux to epigenetic dysregulation.

One of the most extensively studied examples involves mutations in isocitrate dehydrogenase (IDH1 and IDH2). In their wild-type form, IDH enzymes catalyze the oxidative decarboxylation of isocitrate to α-ketoglutarate (α-KG) with concomitant NADPH production. However, recurrent cancer-associated mutations confer a neomorphic enzymatic activity whereby mutant IDH reduces α-KG to D-2-hydroxyglutarate (2-HG) in an NADPH-dependent reaction. Accumulation of 2-HG leads to competitive inhibition of α-KG–dependent dioxygenases, including TET family DNA demethylases and Jumonji-domain histone demethylases, resulting in widespread epigenetic dysregulation, impaired cellular differentiation, and oncogenic transformation [76]. Thus, mutant IDH promotes tumorigenesis not through altered energy production but through metabolite-driven epigenetic remodeling.

Likewise, loss-of-function mutations in succinate dehydrogenase (SDH) or fumarate hydratase (FH) disrupt normal TCA cycle flux and lead to intracellular accumulation of succinate and fumarate, respectively. SDH deficiency, frequently observed in paragangliomas and pheochromocytomas, impairs the conversion of succinate to fumarate and simultaneously compromises complex II of the electron transport chain [77,78]. Generally, elevated succinate and fumarate act as oncometabolites by inhibiting prolyl hydroxylase domain (PHD) enzymes responsible for hydroxylation and degradation of hypoxia-inducible HIF. Inhibition of PHD enzymes results in stabilization of HIF-1α under normoxic conditions, producing a state of “pseudohypoxia” that promotes angiogenesis, glycolysis, and cell survival. In addition, fumarate can covalently modify cysteine residues in proteins through a process known as succination, further altering redox signaling and stress responses. These examples illustrate that mitochondrial alterations in cancer are not merely degenerative defects but represent active metabolic rewiring events that generate bioactive metabolites capable of driving transcriptional, epigenetic, and signaling changes, directly linking to the framework presented in Figure 1. By coupling metabolic flux to gene regulation and oncogenic signaling pathways such as mammalian target of rapamycin (mTOR) and HIF, TCA cycle dysregulation supports tumor growth while creating metabolic liabilities that may be therapeutically exploitable [42,79].

### 4.2. Lipid Metabolism Alterations

Lipid metabolism is another major pathway reprogrammed in cancer cells, with de novo fatty acid synthesis considered a hallmark of tumorigenesis [80]. Newly synthesized fatty acids contribute to membrane biogenesis, energy storage, and cell signaling. Changes in membrane lipid composition influence chemotherapy resistance, as increased lipid saturation and cholesterol content enhance membrane rigidity and reduce drug permeability and oxidative stress. To maintain membrane plasticity, cancer cells rely on lipid desaturation, a process that increases membrane fluidity and supports migration, invasion, and survival [81–83].

Stearoyl-CoA desaturase 1 (SCD1) is a key regulator of lipid desaturation, converting saturated fatty acids into monounsaturated fatty acids and preserving membrane fluidity, cellular signaling, and gene expression. Elevated SCD1 expression is observed in multiple malignancies and correlates with aggressive tumor behavior, poor prognosis, and resistance to chemotherapy [84]. Fatty acid synthase (FASN), the rate-limiting enzyme in lipogenesis, is frequently upregulated through AKT- and HIF-1–mediated signaling under hypoxic conditions, facilitating adaptation to the tumor microenvironment [85]. Therapeutic targeting of FASN has shown promise, particularly in breast cancer and brain metastases [86]. However, the functional role of fatty acid reprogramming remains context-dependent, as cancer cells may rely on both endogenous lipid synthesis and exogenous lipid uptake to sustain growth [87–90].

### 4.3. Amino Acid Metabolism Alterations

Amino acid metabolism is profoundly altered in cancer cells, with glutamine emerging as a central metabolic hub due to the heightened demand imposed by rapid proliferation [91]. Glutamine serves as a critical carbon and nitrogen source for the synthesis of proteins, nucleotides, and other macromolecules, while also fueling the TCA cycle through conversion to glutamate and α-ketoglutarate. In addition, glutamine metabolism supports redox homeostasis by contributing to glutathione synthesis, thereby protecting cancer cells from oxidative stress associated with elevated metabolic activity [91].

Cancer cells can acquire glutamine through unconventional nutrient-scavenging mechanisms. For example, oncogenic RAS signaling promotes extensive cytoskeletal remodeling and the formation of membrane ruffles, which can non-specifically engulf extracellular fluid and its contents, a process known as macropinocytosis [48,92]. This internalized material is delivered to lysosomes for degradation, releasing amino acids like glutamine into the cytoplasm. This mechanism allows RAS-driven cancers, such as pancreatic ductal adenocarcinoma, to sustain tumor growth even under nutrient-limited conditions by effectively utilizing extracellular protein as a nutrient source [48,92]. Targeting glutamine metabolism has therefore emerged as a promising therapeutic strategy, as glutamine deprivation can disrupt tumor metabolism and modulate antitumor immunity, potentially overcoming immune escape mechanisms [93].

Beyond glutamine, cancer cells exhibit increased uptake and utilization of multiple amino acids to support biosynthesis and signaling. Serine and glycine are essential for nucleotide synthesis, protein production, and maintenance of redox balance [94–96]. Tryptophan metabolism is frequently dysregulated in cancer, with enhanced flux through the kynurenine pathway contributing to immunosuppression and immune evasion [97]. Uptake of branched-chain amino acids (BCAAs), particularly leucine, supports tumor growth by activating the mTOR signaling pathway, a key regulator of cell growth and protein synthesis [98]. Furthermore, alterations in the urea cycle enable cancer cells to recycle toxic ammonia for amino acid and nucleotide biosynthesis, further supporting proliferative and invasive behavior [99].

Collectively, these alterations in amino acid metabolism contribute to the metabolic flexibility of cancer cells and provide essential building blocks and energy for tumor progression [100,101]. Therapeutic strategies targeting glutamine metabolism, amino acid transporters, or associated enzymatic pathways represent promising avenues for selectively impairing tumor growth while minimizing effects on normal tissues.

## 5. Molecular Mechanisms Driving Metabolic Reprogramming

Oncogenes act as central drivers of cellular growth and metabolic activity. MYC enhances the expression of glycolytic enzymes, glutamine transporters, and nucleotide synthesis pathways, thereby driving multiple anabolic processes in parallel [102]. AKT increases glucose uptake and stimulates glycolysis, reinforcing the high-energy, high-biomass demands of proliferating tumor cells [103]. Under hypoxic conditions, HIF-1α becomes stabilized and upregulates glycolytic enzymes and PDK1, the latter of which inhibits pyruvate dehydrogenase activity, thereby limiting pyruvate entry into the TCA cycle and further shifting metabolism toward glycolysis [104]. In contrast, tumor suppressors apply brakes to these metabolic programs. p53 suppresses glycolysis while promoting oxidative phosphorylation, and the LKB1/AMPK axis inhibits anabolic pathways when cellular energy levels are low [105,106].

These molecular mechanisms give rise to the three core adaptive pillars of the cancer metabolic ecosystem illustrated in Figure 1: metabolic plasticity, oncometabolite production, and immune-metabolic crosstalk. The first pillar, plasticity, enables cancer cells to dynamically switch between fuel sources, glycolysis, OXPHOS, glutaminolysis, or fatty acid oxidation, in response to environmental stress or therapeutic pressure. The second pillar, oncometabolite production, creates a direct link between metabolic flux and epigenetic control, as discussed in Section 4. The third pillar, explored below, involves the complex interplay between cancer cells and the immune microenvironment.

The TME adds another dimension of complexity, functioning as an ecosystem in which cancer cells, stromal cells, immune cells, and the extracellular matrix continuously interact. These interactions foster both metabolic competition and metabolic cooperation. Cancer cells often outcompete T cells for glucose, impairing T cell function and facilitating immune evasion; they also consume key amino acids such as arginine and tryptophan, further weakening anti-tumor immunity [107,108]. This immune-metabolic crosstalk has profound therapeutic implications. The high glycolytic flux of tumors depletes glucose and generates a lactate-rich, acidic microenvironment that impairs T-cell proliferation and effector function while promoting regulatory T-cell (Treg) stability. Furthermore, cancer cells consume or degrade key amino acids like arginine (via arginase) and tryptophan (via IDO1), which are essential for T-cell activation and survival. Tryptophan depletion, coupled with the accumulation of immunosuppressive kynurenine, drives T-cell anergy and promotes Treg differentiation. This mechanistic insight has catalyzed the development of immuno-metabolic combination strategies.

In some settings, metabolic crosstalk creates symbiotic relationships, most notably in the “reverse Warburg effect,” where cancer-associated fibroblasts adopt a glycolytic phenotype and secrete lactate that tumor cells utilize for oxidative phosphorylation [109]. Hypoxic regions within the TME stabilize HIF-1α, triggering a metabolic shift toward glycolysis and angiogenesis that promotes tumor aggressiveness, adaptation, and resistance to therapy [110]. Interestingly, the specific oncogenic driver profoundly shapes the resulting metabolic phenotype. For example, KRAS-mutant cancers (e.g., PDAC, lung adenocarcinoma) often upregulate macropinocytosis and glutamine metabolism to fuel growth in nutrient-poor environments. In contrast, MYC-driven cancers (e.g., Burkitt lymphoma, neuroblastoma) are characterized by coordinated upregulation of glycolysis, glutaminolysis, and nucleotide synthesis. Tumors with loss of von Hippel-Lindau (VHL) (e.g., ccRCC) exhibit pseudohypoxic states with constitutive HIF activation, driving glycolysis and lipid storage. This oncogene-specific metabolic rewiring creates distinct vulnerabilities that may be therapeutically exploited.

## 6. Therapeutic Implications of Immune-Metabolic Crosstalk

The intersection of cancer metabolism and antitumor immunity represents a critical frontier with significant therapeutic implications. Metabolic interventions can have dual, and sometimes opposing, effects on immune cell function, necessitating careful consideration in combination strategies.

The high glycolytic demand of tumors creates a glucose-depleted, lactate-rich microenvironment that disrupts T-cell glycolytic metabolism and effector function. Tumor-derived lactate suppresses T-cell proliferation and cytokine production while promoting regulatory T-cell (Treg) differentiation and M2-like macrophage polarization [107,108]. This metabolic barrier may limit the efficacy of immune checkpoint inhibitors (ICIs), particularly in highly glycolytic tumors. Preclinical studies suggest that reducing tumor glycolytic flux via LDHA or MCT4 inhibition can alleviate lactate-mediated immunosuppression and enhance ICI responses [109]. However, caution is warranted, as activated T cells themselves rely on aerobic glycolysis, and systemic glycolytic inhibition could inadvertently impair antitumor immunity.

Cancer cells and immune cells also compete for essential amino acids within the TME. Arginine depletion by tumor-associated myeloid cells (via arginase) suppresses T-cell proliferation and function, while tryptophan catabolism via IDO1 generates immunosuppressive kynurenines that promote T-cell anergy and Treg differentiation [97]. Clinical strategies targeting IDO1 have shown limited efficacy in phase III trials as monotherapies, tempering early enthusiasm for this approach, but are being explored in combination with ICIs. Similarly, glutamine metabolism presents a double-edged sword: GLS1 inhibition with telaglenastat may reduce glutamine availability for tumor growth; however, activated T cells also depend on glutamine for proliferation and differentiation into effector subsets [93]. Ongoing trials combining telaglenastat with nivolumab (NCT02771626) will help determine whether metabolic priming can enhance or impair immune responses. Along these lines, emerging evidence suggests that oncometabolites such as 2-HG may directly influence immune cell function beyond their effects on cancer cell epigenetics. 2-HG has been shown to impair T-cell activation and promote an immunosuppressive TME in IDH-mutant tumors (see Enasidenib and Ivosidenib in the coming sections). This raises the possibility that IDH inhibitors may enhance antitumor immunity by both reducing 2-HG levels in cancer cells and relieving direct immunosuppression of T cells, an area warranting further investigation.

Successful immuno-metabolic combinations will require a nuanced understanding of the differential metabolic dependencies of cancer cells versus specific immune subsets within the TME. Strategies that target metabolic pathways uniquely essential for tumor cells but dispensable for effector T cells (e.g., MCT4, which is frequently overexpressed in highly glycolytic cancer cells and less critical in effector T cells) may offer a therapeutic window. Alternatively, metabolic interventions that remodel the TME to be less hostile (e.g., reducing lactate, alleviating nutrient competition within the TME) may synergize with ICIs regardless of direct effects on immune cell metabolism. Addressing these questions will be critical for translating metabolic therapies into effective immuno-oncology combinations.

## 7. Therapeutic Targeting of Cancer Metabolism

The distinctive metabolic dependencies of cancer cells provide multiple avenues for therapeutic intervention, many of which are now being actively explored in preclinical and clinical settings. Glycolysis remains one of the most intensively targeted pathways. Inhibitors of early glycolytic steps, such as hexokinase 2 (HK2), including 2-deoxyglucose, benitrobenrazide, and 5-thio-D-glucose, aim to block the conversion of glucose to glucose-6-phosphate and suppress glycolytic flux. However, despite decades of study, 2-DG’s clinical development has been limited by poor bioavailability, a narrow therapeutic window requiring very high doses for efficacy, and significant toxicities like glycemia and cardiac effects, limiting its clinical advancement beyond early-phase trials. At the level of glucose uptake, several glucose transporter (GLUT) inhibitors, such as BAY-876, WZB117, STF-31 (originally characterized as a GLUT1 inhibitor but later shown to also inhibit nicotinamide phosphoribosyltransferase), and fasentin, have been developed to reduce the elevated glucose import characteristic of highly glycolytic tumors. Specifically, BAY-876 is a potent and selective GLUT1 inhibitor with an IC_50_ in the low nanomolar range, but remains in the preclinical phase of development. Other agents like WZB117 have demonstrated in vivo efficacy in xenograft models but have not yet entered clinical trials. Downstream, inhibition of lactate dehydrogenase A (LDHA) with experimental agents such as oxamate disrupts pyruvate–lactate interconversion and limits lactate production, a key feature of the Warburg phenotype.

Amino acid metabolism represents another major vulnerability in cancer. Many tumors exhibit increased uptake of neutral amino acids through transporters such as ASCT2 and LAT1; inhibitors, including V-9302, SN40, BCH, and KMH-233, are designed to restrict amino acid availability and impair biosynthetic and signaling pathways. Glutamine addiction is particularly widespread, and targeting glutaminase (GLS) with inhibitors such as BPTES, telaglenastat (CB-839) [111], and IPN60090 reduces glutamine conversion to glutamate, thereby limiting anaplerosis and redox balance. Clinically validated amino acid depletion strategies, such as L-asparaginase in leukemias and arginine deiminase in arginine-auxotrophic tumors, further underscore the therapeutic relevance of this approach [112].

Mitochondrial metabolism has also emerged as a promising target. Inhibition of mitochondrial complex I [113] using agents such as IACS-010759, DX2–201, or metformin interferes with oxidative phosphorylation and ATP production. In parallel, mutant isocitrate dehydrogenase 1 and 2 (mIDH1/2) enzymes—responsible for the aberrant production of the oncometabolite 2-hydroxyglutarate—are successfully targeted by enasidenib and ivosidenib, which are approved or under active clinical investigation [114]. Fatty acid oxidation can be suppressed by targeting carnitine palmitoyltransferase 1 (CPT1) with etomoxir, thereby limiting mitochondrial import of long-chain fatty acids.

Lipid biosynthesis and uptake constitute additional therapeutic entry points. FASN inhibitors such as denifanstat (TVB-2640), UCM05, and trans-C75 block de novo lipid synthesis required for membrane production and signaling in proliferating cancer cells. Upstream, acetyl-CoA carboxylase (ACC) inhibitors, including soraphen A and ND-646, restrict malonyl-CoA formation and fatty acid synthesis. Lipid uptake is being explored as a target through anti-CD36 strategies, although clinical validation remains limited [115]. Furthermore, blocking lactate transport through monocarboxylate transporters (MCT1 and MCT4) using agents such as AZD3965, BAY-8002, and syrosingopine disrupts metabolic symbiosis between glycolytic and oxidative tumor cell populations.

Metabolic signaling nodes that integrate nutrient availability and growth control are also therapeutically relevant. Inhibition of mechanistic target of rapamycin complex 1 (mTORC1) with agents such as temsirolimus interferes with nutrient sensing and protein synthesis, while targeting phosphofructokinase-2 (PFK-2) using compounds such as PFK-015 modulates fructose-2,6-bisphosphate levels and fine-tunes glycolytic flux.

Despite these advances, targeting cancer metabolism presents several challenges. Tumors exhibit significant metabolic plasticity and heterogeneity, enabling them to switch fuel sources or activate compensatory pathways that confer therapeutic resistance [116,117]. Toxicity is another concern, as many targeted pathways are essential for normal cellular function, raising the risk of off-target effects. Furthermore, the complex metabolic interplay within the TME can blunt the efficacy of metabolic drugs [118].

Future strategies are expected to address these barriers. Combination therapies, pairing metabolic inhibitors with chemotherapy, radiation, or immunotherapy, hold promise for overcoming resistance and enhancing treatment responses [119,120]. Personalized medicine approaches, including metabolic profiling, may help identify patient-specific vulnerabilities that can guide targeted interventions [121]. Dietary interventions such as caloric restriction or ketogenic diets are being explored as adjunctive strategies to impose metabolic stress on tumor cells [122]. Advances in computational modeling and artificial intelligence may further accelerate progress by mapping metabolic networks, predicting adaptive responses, and uncovering novel targets or synthetic lethal interactions [123,124].

## 8. Specific Agents

**Figure 3.**
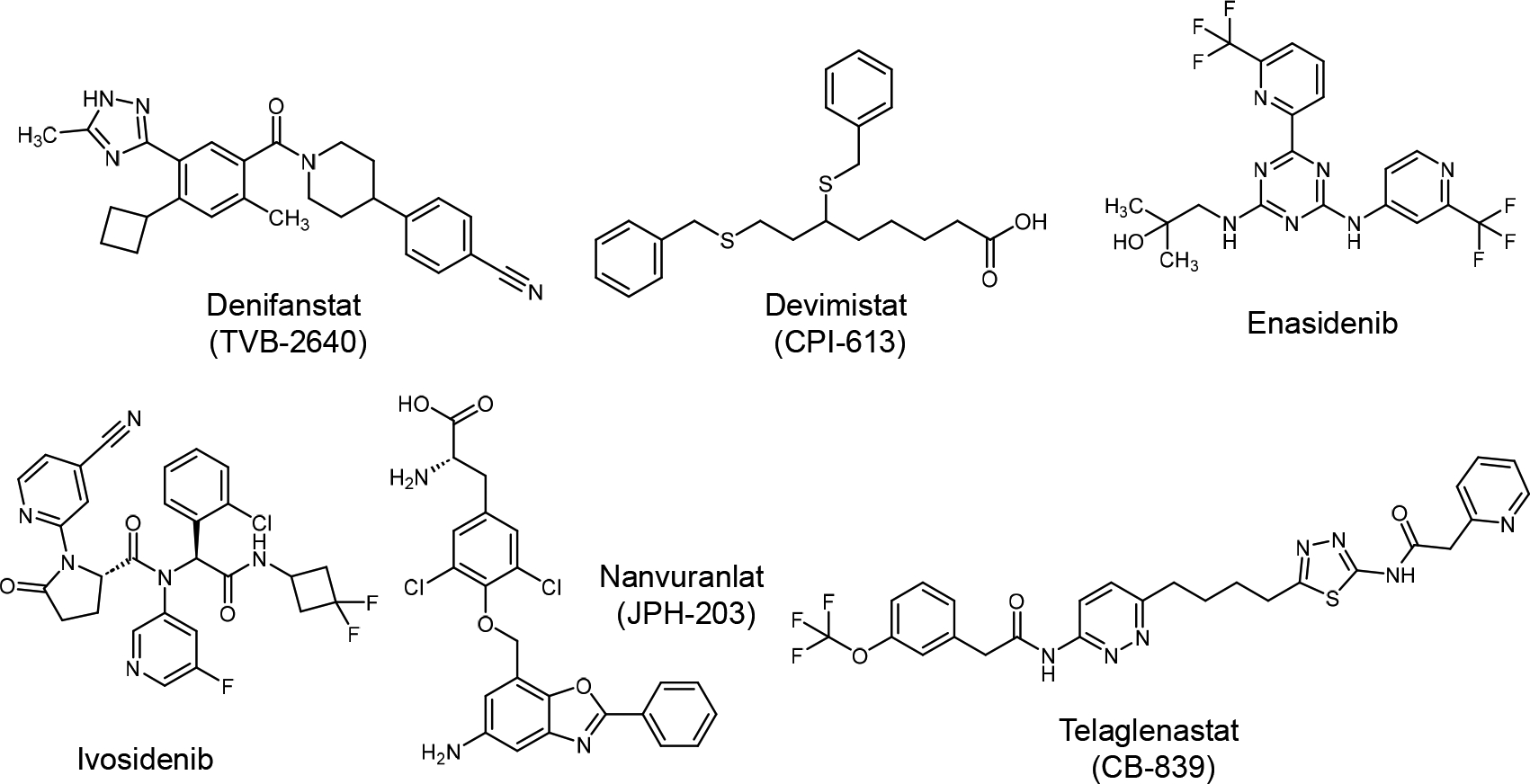
The chemical structures of some of the drugs that affect the metabolism of cancer cells.

We discuss below specific agents (Figure 3 and Table 1); however, more agents with relevant information (IC_50_ values, clinical trial phases, and side effects) are tabulated and submitted as Supplementary Materials.

### 8.1. Denifanstat (TVB-2640)

Denifanstat (TVB-2640) is a first-in-class, oral, and selective inhibitor of fatty acid synthase (FASN), a metabolic enzyme critical for the de novo synthesis of fatty acids [125]. It inhibits FASN with an IC_50_ of 0.052 μM and an EC_50_ of 0.072 μM. In many aggressive cancers, tumor cells become dependent on this endogenous lipogenesis pathway to fuel rapid proliferation, maintain survival signaling, and develop resistance to therapies [126]. By blocking FASN, denifanstat aims to induce a state of “metabolic synthetic lethality,” selectively starving cancer cells of the essential lipids they require to grow and survive while sparing normal cells [127].

The clinical development of denifanstat in oncology has progressed across a range of solid tumors where this metabolic vulnerability is pronounced. Its most advanced application is in recurrent high-grade astrocytoma and glioblastoma, where it is being evaluated in a Phase 2 clinical program evaluating its combination with bevacizumab in recurrent high-grade gliomas, based on earlier evidence of improved disease control [128]. Parallel clinical programs are investigating its potential in other difficult-to-treat cancers, including KRAS-mutant non-small cell lung cancer, metastatic castration-resistant prostate cancer, and HER2-positive breast cancer in combination with targeted therapies [129–131]. This broad investigational strategy seeks to validate FASN inhibition as a novel, targeted metabolic approach for disrupting a fundamental pathway in tumor biology across multiple cancer types.

### 8.2. Devimistat (CPI-613)

Devimistat (CPI-613) is a first-in-class, lipoate-mimetic agent that selectively targets the mitochondrial enzymes pyruvate dehydrogenase and α-ketoglutarate dehydrogenase, which are critical hubs of central carbon metabolism in cancer cells [129]. Rather than functioning as a classical active-site inhibitor, it induces metabolic stress through redox modulation of these mitochondrial enzyme complexes, leading to impaired TCA cycle flux. Although functional inhibition appears sustained in preclinical systems, definitive evidence of irreversible covalent modification of the native lipoate cofactor remains limited. Nevertheless, this unique mechanism disrupts both the TCA cycle and the subsequent flow of electrons to the mitochondrial electron transport chain, leading to energy stress and cancer cell death, particularly in metabolically vulnerable tumors [132,133]. By targeting these mitochondrial pathways, devimistat exploits the altered metabolism of tumors, which are often more dependent on mitochondrial function for growth and survival than normal cells. While not entirely selective, its anti-cancer activity is hypothesized to stem from cancer cells’ heightened dependence on these mitochondrial hubs for both energy production (TCA cycle) and biosynthesis, potentially offering a therapeutic window over normal cells, which may be more metabolically flexible [132,133].

The clinical development of devimistat in oncology has been extensive, focusing on aggressive and treatment-resistant cancers. Its most advanced and impactful trials have been in hematologic malignancies and pancreatic cancer. In acute myeloid leukemia, a pivotal Phase III trial (NCT03504410) evaluated devimistat in combination with high-dose cytarabine and mitoxantrone in patients with relapsed/refractory AML, although the trial was ultimately halted for futility and did not demonstrate a significant survival benefit [134]. In solid tumors, a landmark Phase III trial (NCT03504423) investigated devimistat combined with modified FOLFIRINOX (mFFX) versus FOLFIRINOX alone for first-line metastatic pancreatic adenocarcinoma. Although the primary endpoint of overall survival was not met, the combination showed a significant improvement in progression-free survival and objective response rate, supporting its continued investigation [135–137]. Additional clinical trials have explored devimistat across a wide spectrum of other cancers, including relapsed/refractory Burkitt lymphoma (NCT03793140), small cell lung cancer (NCT01931787), biliary tract cancer (NCT04203160), T-cell lymphomas (NCT02168140, NCT04217317), and clear cell sarcoma (NCT04593758), underscoring its broad therapeutic potential as a novel metabolic disruptor in oncology [138–143].

### 8.3. Enasidenib and Ivosidenib

Enasidenib and Ivosidenib are first-in-class, targeted oral inhibitors of mutant isocitrate dehydrogenase (IDH) enzymes, representing a paradigm shift in the treatment of AML and related hematologic malignancies. Enasidenib specifically inhibits the mutant IDH2 enzyme, while Ivosidenib targets mutant IDH1 [144]. In normal cells, IDH enzymes catalyze the conversion of isocitrate to alpha-ketoglutarate (α-KG). Mutations in IDH1 or IDH2, found in approximately 20% of AML cases, confer a neomorphic activity that leads to the production of the oncometabolite D-2-hydroxyglutarate (2-HG) [145]. This aberrant metabolite competitively inhibits α-KG-dependent dioxygenases, including those involved in DNA and histone demethylation, resulting in a block in cellular differentiation and promotion of leukemogenesis [146]. By selectively binding to the mutant enzyme, both inhibitors dramatically reduce 2-HG levels, releasing the differentiation block and allowing for the maturation of leukemic blasts into functional myeloid cells, a mechanism distinct from traditional cytotoxic chemotherapy [147].

The clinical development of both agents is anchored in pivotal trials leading to regulatory approvals. For Ivosidenib, the single-arm AG120-C-001 study (NCT02074839) demonstrated efficacy in patients with relapsed/refractory (R/R) IDH1-mutant AML, resulting in a complete remission (CR) or CR with partial hematologic recovery (CRh) rate of 30.4%, leading to its FDA approval in 2018 [148]. Its role was expanded to newly diagnosed IDH1-mutant AML patients ineligible for intensive chemotherapy based on the AG120 study (NCT02677922), where Ivosidenib plus azacitidine significantly improved event-free survival compared to placebo plus azacitidine [149]. Similarly, Enasidenib was approved for R/R IDH2-mutant AML based on results from the AG221–001 study (NCT01915498), which reported a CR rate of 19.3% and a median overall survival of 9.3 months [150]. A confirmatory randomized Phase III trial (IDHENTIFY, NCT02577406) compared Enasidenib to conventional care regimens in older patients with R/R AML; while it did not meet its primary survival endpoint, it validated the agent’s clinical activity and differentiation syndrome as a class-effect toxicity [151]. A key indication for Ivosidenib in solid tumors was established by the ClarIDHy trial (NCT02989857), a randomized Phase III study in previously treated advanced IDH1-mutant cholangiocarcinoma, which demonstrated a significant improvement in progression-free survival over placebo, leading to its approval in this setting [152]. Ongoing research now explores its potential in the adjuvant/curative setting for cholangiocarcinoma (e.g., NCT04195555) and in other solid tumors like chondrosarcoma (NCT04278781) [153,154]. Furthermore, extensive combinatorial strategies are being evaluated, such as combining these inhibitors with standard induction chemotherapy (NCT02632708), hypomethylating agents (NCT02677922), BCL-2 inhibitors (e.g., venetoclax; NCT04092179, NCT04774393) [155], and FLT3 inhibitors (e.g., gilteritinib; NCT05756777), to deepen and prolong responses in both frontline and relapsed settings of AML and other myeloid neoplasms.

### 8.4. Nanvuranlat (JPH-203)

Nanvuranlat (JPH-203) is a first-in-class, highly selective, small-molecule inhibitor targeting the L-type amino acid transporter 1 (LAT1; SLC7A5). LAT1 is a high-affinity transporter of large neutral essential amino acids, including leucine, isoleucine, phenylalanine, and tyrosine, and functions as a heterodimer with the heavy chain CD98 (SLC3A2) to facilitate membrane localization and activity. By mediating leucine uptake, LAT1 plays a central role in activating mTORC1 through amino acid–sensing pathways, thereby promoting protein synthesis, anabolic growth, and tumor cell proliferation [156]. LAT1 expression is markedly upregulated in a wide range of malignancies and is frequently associated with high-grade tumors and poor prognosis. Its overexpression is driven by oncogenic signaling networks, including MYC-mediated transcriptional programs that enhance nutrient transporter expression to meet anabolic demand. In hypoxic tumor regions, HIF-dependent signaling, particularly via HIF2α, can further induce SLC7A5 expression, linking amino acid uptake to adaptive metabolic responses and sustained mTORC1 activation under stress conditions. Through this integration of nutrient transport with oncogenic and hypoxic signaling, LAT1 functions as a metabolic gatekeeper that enables tumor cells to maintain anabolic growth despite microenvironmental constraints, making it an attractive and mechanistically rational therapeutic target [156,157]. By competitively inhibiting LAT1-mediated amino acid influx, nanvuranlat disrupts these fundamental oncogenic pathways, inducing cell cycle arrest and apoptosis specifically in cancer cells with minimal effects on normal tissues [158].

The clinical development of nanvuranlat has focused primarily on advanced biliary tract cancers (BTC), a malignancy with high unmet need and frequent LAT1 overexpression. A pivotal Phase 1 study in Japanese patients with advanced solid tumors (JapicCTI-173,741) established the safety, pharmacokinetics, and recommended Phase 2 dose, while providing preliminary evidence of antitumor activity, particularly in BTC patients [159]. This supported the design of a subsequent Phase 2a study (NCT04306367) specifically in advanced, refractory biliary tract cancer, which demonstrated promising efficacy signals, including disease control and manageable tolerability [160]. Based on this foundation, the global KEYNOTE-B98/J1G-MC-JZJC trial (NCT05102022) was initiated. This study consists of two parts: Part A, a dose-finding and safety run-in of nanvuranlat in combination with pembrolizumab and gemcitabine/cisplatin, and Part B, a randomized Phase 3 component comparing this triplet combination to the standard-of-care gemcitabine/cisplatin regimen in treatment-naïve patients with advanced BTC. This trial represents the most advanced clinical evaluation of nanvuranlat, aiming to validate LAT1 inhibition as a novel therapeutic strategy for this aggressive cancer [161].

### 8.5. Telaglenastat (CB-839)

Telaglenastat (CB-839) is a first-in-class, potent, selective, and orally bioavailable inhibitor of glutaminase 1 (GLS1), the enzyme responsible for converting glutamine to glutamate in the first step of glutaminolysis [162]. Many cancer cells exhibit a profound dependence on glutamine to fuel anabolic growth, maintain redox balance, and support mitochondrial metabolism—a state known as “glutamine addiction” [29]. By disrupting this critical metabolic pathway, Telaglenastat induces metabolic stress, inhibits tumor growth, and can synergize with a variety of targeted therapies and chemotherapies, particularly in tumors with specific genetic vulnerabilities (e.g., KEAP1/NRF2 mutations) that render them more reliant on glutamine metabolism [163].

The clinical development of Telaglenastat has been broad, investigating its safety and efficacy across numerous solid tumors and hematologic malignancies, both as a monotherapy and in combination regimens. Key trials have highlighted its potential in specific oncologic contexts. In ccRCC, the randomized, double-blind CANTATA trial (NCT03428217) evaluated Telaglenastat plus cabozantinib versus placebo plus cabozantinib in previously treated patients. While the study did not meet its primary endpoint of improved progression-free survival in the overall population, a prespecified subgroup analysis suggested potential benefit in patients with specific metabolic profiles, underscoring the need for biomarker-driven patient selection [164–171]. In non-small cell lung cancer (NSCLC), significant research has focused on tumors with KEAP1/NRF2 mutations, which confer a poor prognosis and heightened glutamine dependence. The KEAPSAKE trial (NCT04265534) evaluated Telaglenastat in combination with standard first-line chemoimmunotherapy in this molecular subset but was terminated early due to futility in improving progression-free survival [164]. Other notable combination trials include investigating Telaglenastat with everolimus in RCC (NCT03163667), Nivolumab in melanoma, RCC, and NSCLC (NCT02771626), and Talazoparib in solid tumors (NCT03875313), among others [167–171]. While definitive registration trials have yet to confirm a major survival benefit in unselected populations, the extensive clinical dataset continues to inform the pursuit of predictive biomarkers to identify patients most likely to respond to this novel metabolic therapy.

## 9. Translational Challenges and Clinical Limitations

Although targeting cancer metabolism remains conceptually attractive, clinical translation has proven far more complex than initially anticipated. Several metabolic inhibitors that demonstrated strong preclinical efficacy have failed to produce durable survival benefits in late-phase clinical trials, highlighting intrinsic biological constraints within tumor metabolic networks [116–118,135,164]. As depicted in the lower tier of Figure 1, these clinical limitations arise from three interconnected barriers: metabolic redundancy and adaptive resistance, microenvironmental buffering, and profound patient heterogeneity.

A principal challenge is metabolic plasticity. Unlike oncogenic driver mutations that may create relatively fixed signaling dependencies, metabolic programs are dynamic and responsive to environmental and therapeutic pressures. When dominant pathways such as glycolysis or glutaminolysis are pharmacologically inhibited, tumor cells frequently compensate by activating alternative fuel sources, including fatty acid oxidation, acetate metabolism, serine/glycine pathways, or enhanced oxidative phosphorylation [116,117]. For instance, glutaminase inhibition with telaglenastat showed promising metabolic suppression in early-phase studies; however, larger trials such as CANTATA (NCT03428217) and KEAPSAKE (NCT04265534) failed to demonstrate significant progression-free survival advantages in unselected patient populations [164,165]. Preclinical and translational analyses suggest that tumors may compensate through enhanced glucose oxidation and parallel amino acid utilization, reflecting the flexibility of central carbon metabolism [47,93]. Likewise, inhibition of glycolysis can promote mitochondrial reliance, while complex I inhibition may enhance glycolytic flux, illustrating bidirectional metabolic compensation [75,113]. These findings emphasize that cancer metabolism operates as a flexible network that can rapidly rewire under selective pressure.

Beyond plasticity, metabolic redundancy further limits therapeutic durability. Cancer metabolism is highly interconnected, with multiple pathways converging on shared intermediates such as acetyl-CoA, α-ketoglutarate, and NADPH [23,24]. Inhibition of a single enzyme often fails to deplete downstream metabolites because alternative pathways replenish these pools through anaplerotic reactions or salvage mechanisms. NADPH, for example, can be generated via the oxidative branch of the pentose phosphate pathway, malic enzyme activity, or IDH-mediated reactions [64,65]. Acetyl-CoA may derive from glucose, glutamine, acetate, or fatty acids, depending on nutrient availability and oncogenic context [23,24]. Likewise, nucleotide synthesis can proceed through both de novo pathways and salvage routes [58,59]. This network robustness frequently results in cytostatic rather than cytotoxic effects, which may explain why many single-agent metabolic inhibitors demonstrate modest clinical benefit despite strong biochemical target engagement [116,117].

The TME further complicates metabolic targeting through microenvironmental buffering. Tumors exist within a complex metabolic ecosystem composed of stromal fibroblasts, immune cells, adipocytes, and endothelial cells that collectively influence substrate availability and metabolic flux [8,30,107]. Stromal-derived lactate can be oxidized by tumor cells as an alternative carbon source, thereby sustaining growth under glycolytic inhibition [67,68]. Adipocytes in metastatic niches may provide fatty acids that fuel β-oxidation and promote resistance to metabolic stress [53,87]. Fibroblasts can supply amino acids and other metabolites in nutrient-restricted conditions, buffering against pharmacologic blockade of specific pathways [30]. Moreover, hypoxic regions stabilize HIF-1α, reinforcing glycolytic programs and potentially reducing susceptibility to mitochondrial inhibitors [104,110]. These ecosystem-level interactions highlight that metabolic therapies targeting intrinsic tumor pathways alone may be insufficient without consideration of microenvironmental contributions.

A further limitation has been inadequate biomarker-driven patient selection, a critical component of addressing patient heterogeneity. Unlike kinase inhibitors targeting defined mutations, many metabolic inhibitors have been tested in heterogeneous populations without stratification based on metabolic dependency. Evidence suggests that glutaminase inhibition may preferentially benefit tumors harboring KEAP1/NRF2 alterations associated with enhanced glutamine reliance [163]. In contrast, IDH inhibitors demonstrate efficacy only in tumors with specific IDH1 or IDH2 mutations that drive oncometabolite accumulation [144–147]. Lipogenic targeting strategies, including FASN inhibition, appear most relevant in tumors characterized by upregulated fatty acid synthesis [55,125,127]. The absence of robust predictive biomarkers likely contributed to negative or inconclusive outcomes in trials evaluating agents such as Devimistat in pancreatic cancer and Telaglenastat in renal and lung cancers [135,164,165]. Future progress will depend on integrating metabolomic profiling, genomic alterations, and functional imaging to identify metabolically vulnerable subgroups [121].

Systemic toxicity also constrains therapeutic windows. Core metabolic pathways are essential for normal tissue function, including immune cell activation, hematopoiesis, and hepatic metabolism [31–35]. Inhibiting glycolysis, mitochondrial respiration, or amino acid metabolism may therefore affect rapidly proliferating normal cells and metabolically active organs. Furthermore, metabolic inhibition may impair antitumor immunity by disrupting T-cell glycolytic and mitochondrial programming and by exacerbating nutrient competition within the tumor microenvironment [25,70,107]. These systemic considerations underscore the necessity of tumor-selective targeting strategies or context-dependent metabolic interventions.

## 10. Metabolic Heterogeneity in Cancer: Implications for Therapy

A fundamental reason for the limited success of uniform metabolic targeting strategies is the heterogeneity of cancer metabolism. This variability, a core component of the therapeutic barriers in our framework (Figure 1), underscores why broad metabolic inhibition is unlikely to succeed without biomarker-driven patient selection. It exists across tumor types, within individual tumors, and over time in response to therapy.

Inter-tumoral heterogeneity reflects differences in tissue origin, oncogenic drivers, and microenvironmental context [24,25]. IDH-mutant gliomas, for example, accumulate the oncometabolite 2-hydroxyglutarate, which reshapes epigenetic regulation and cellular differentiation [145–147]. Breast and prostate cancers often exhibit lipogenic phenotypes driven by FASN overexpression and altered lipid metabolism [55,86]. Pancreatic cancers demonstrate enhanced nutrient scavenging mechanisms, including macropinocytosis, enabling adaptation to nutrient-poor environments [48,92]. These examples illustrate that metabolic vulnerabilities are highly context-dependent rather than universal features of malignancy [47,116].

Intra-tumoral and spatial heterogeneity further complicate therapeutic strategies. Within a single tumor mass, hypoxic regions preferentially rely on glycolysis, whereas well-perfused areas may depend more heavily on oxidative phosphorylation [36,110]. Subpopulations of cancer stem-like cells have been reported to exhibit increased mitochondrial dependency in certain tumor contexts and metabolic flexibility, contributing to resistance and disease recurrence [116]. This spatial compartmentalization creates metabolic niches that allow distinct subclones to survive selective pressures, thereby facilitating clonal evolution under therapeutic stress [117].

Metabolic phenotypes are also temporally dynamic and therapy-responsive. Chemotherapy, radiation, and targeted therapies can induce metabolic stress that prompts adaptive rewiring [116–118]. In several tumor models, platinum resistance has been associated with shifts toward fatty acid oxidation and altered lipid metabolism [86,87]. Resistance to targeted therapies may correlate with increased mitochondrial respiration or altered redox balance [75]. Immune checkpoint blockade modifies nutrient competition and metabolic programming within the tumor microenvironment, further reshaping metabolic dependencies [107,119]. Thus, metabolism must be viewed as an evolving phenotype rather than a static hallmark.

Given these layers of heterogeneity, future metabolic therapies will require systems-level approaches rather than single-enzyme inhibition. Rational combinations targeting parallel metabolic pathways, synthetic lethality strategies exploiting mutation-specific vulnerabilities, and metabolomic-guided patient stratification represent promising directions [119–121,127]. Integration of computational modeling and artificial intelligence may further enable prediction of adaptive rewiring and therapeutic resistance [123,124]. Precision metabolic oncology will therefore require defining not only a tumor’s genomic profile but also its functional metabolic phenotype within its ecological context.

Together, while cancer metabolism remains a compelling therapeutic target, its clinical exploitation requires recognition of plasticity, redundancy, and heterogeneity as defining biological principles rather than anomalies [23–25,116,117]. Progress will depend on biomarker-guided patient selection, rational combination strategies, and ecosystem-level understanding of tumor metabolic networks.

## 11. Outstanding Questions and Future Directions

Despite significant progress, the framework presented in Figure 1 highlights several critical unanswered questions. First, what are the temporal dynamics of metabolic plasticity? How do tumors transition between glycolytic and oxidative states during progression, metastasis, and therapeutic intervention? Second, can we therapeutically target the crosstalk between oncometabolites and the immune microenvironment? For example, does 2-HG directly impair T-cell function independent of its effects on cancer cell epigenetics? Third, what is the optimal way to integrate metabolic therapies with immunotherapy? Does inhibiting glutamine metabolism enhance T-cell function by relieving nutrient competition, or does it paradoxically impair T-cell activation and expansion? Fourth, can we identify metabolic pathways that are differentially essential in cancer cells versus antitumor immune cells to design combinations that enhance, rather than impair, immunotherapy responses? Answering these questions will require moving beyond static metabolic snapshots toward dynamic, in vivo models capable of capturing flux, cellular interaction, and the evolving metabolic ecosystem.

## 12. Conclusions

Cancer metabolism has evolved from a historical biochemical observation into a central organizing principle in modern oncology. As summarized in the framework presented in Figure 1, the metabolic rewiring of core pathways is now recognized as a dynamic ecosystem driven by genetic and environmental pressures. This ecosystem is defined by three interconnected adaptive pillars: metabolic plasticity, which enables fuel switching under stress; oncometabolite production, which couples metabolic state to epigenetic gene expression; and immune-metabolic crosstalk, which governs immune evasion and therapeutic response. Through coordinated alterations in glycolysis, mitochondrial function, amino acid utilization, lipid synthesis, and redox regulation, cancer cells secure the energetic resources, biosynthetic precursors, and oxidative balance necessary to sustain proliferation, survival, and metastatic progression.

However, as the lower tier of Figure 1 illustrates, the clinical translation of metabolic targeting has revealed formidable barriers. Tumor metabolism is neither linear nor static; it is adaptive, redundant, and profoundly heterogeneous. Metabolic plasticity enables dynamic fuel switching under therapeutic pressure, while network redundancy and microenvironmental buffering protect against single-pathway inhibition. Inter- and intra-tumoral heterogeneity further complicates the identification of universal metabolic vulnerabilities. These biological features help explain why many promising metabolic inhibitors have demonstrated limited efficacy in unselected clinical populations, as exemplified by the failures of Telaglenastat in unselected renal cancer and Devimistat in pancreatic cancer.

The next phase of progress in cancer metabolism will require a shift from reductionist enzyme targeting toward systems-level precision strategies that embrace the complexity of the framework. This shift will be driven by the integration of emerging technologies that are poised to transform the field. Spatial metabolomics and transcriptomics will enable in situ mapping of metabolic heterogeneity at single-cell resolution [172,173]. Single-cell metabolic profiling approaches such as SCENITH and single-cell metabolomics will further resolve phenotypic diversity within tumor and immune populations [174,175]. In parallel, AI-driven metabolic network modeling and machine learning will integrate multi-omics datasets to predict adaptive resistance and identify synthetic lethal interactions tailored to individual metabolic ecosystems [176–178].

Rational combination therapies designed to preempt adaptive rewiring, along with biomarker-guided patient stratification, will likely determine whether metabolic interventions achieve durable clinical benefit. Moreover, understanding the metabolic interplay between tumor cells and the immune microenvironment may unlock synergistic opportunities with immunotherapy, targeting the third pillar of our framework.

Rather than diminishing its therapeutic promise, recent challenges have clarified the complexity of cancer metabolism and refined its translational trajectory. By embracing metabolic heterogeneity as a defining biological feature and leveraging emerging technologies to map dynamic metabolic states, the field is poised to transform foundational metabolic principles into precise, effective, and personalized anticancer therapies.

## Supplementary Material

Suppl Info

**Supplementary Materials:** The following supporting information can be downloaded at: https://www.mdpi.com/article/10.3390/ddc5010017/s1, Table S1: Specific Candidates [125,134,135,147,150,162,164,179–194].

## Figures and Tables

**Table 1.** Selected Anti-Cancer Drugs Targeting Altered Metabolic Pathways

	Function	Candidate Drugs
Alanine-Serine-Cysteine Transporter 2 (ASCT2)	Transport neutral amino acids	V9302, SN40, SN02
Acetyl-CoA Carboxylase (ACC)	Catalyze the carboxylation of acetyl-CoA to produce malonyl-CoA	Soraphen A, Andrimid, TOFA, Firsocostat (ND-646), CP-640186, MEDICA 16
Carnitine Palmitoyltransferase 1 (CPT1)	Catalyze the transport of long-chain fatty acids into mitochondria	Etomoxir
Fatty Acid Synthase (FASN)	Synthesize fatty acid	Denifanstat (TVB-2640), UCM05, Trans-C75
Glucose transporters (GLUTs)	Transport glucose	BAY-876, WZB117, STF-31, Fasentin, GLUT4-IN-2
Glutaminase (GLS)	Metabolize glutamine into glutamate	BPTES, Telaglenastat (CB-839), LWG-301, IPN60090
Hexokinase 2 (HK2)	Converts glucose to glucose-6-phosphate to start glycolysis	2-Deoxyglucose, Benitrobenrazide, 5-Thio-D-glucose (5TG)
Lactate dehydrogenase A (LDH A)	Catalyzes the conversion of pyruvate to lactate and back	Oxamate
L-Type Amino Acid Transporter 1 (LAT1)	Transport neutral amino acids	BCH, KMH-233, GPNA hydrochloride, Nanvuranlat (JPH203)
Mitochondrial Complex I (MC I)	The major entry point for electrons into the respiratory chain	IACS-010759, DX2–201, HQNO, SCAL-266, Metformin
Monocarboxylate Transporter 1 (MCT1)	Transport and import lactate	BAY-8002, AZD3965
Monocarboxylate Transporter 4 (MCT4)	Transport and export lactate	VB124, Syrosingopine, AZD0095
Mutant Isocitrate Dehydrogenase 1 and 2 (mIDH 1/2)	Enzymes arise from genetic mutations, which change their normal function to produce an “oncometabolite,” 2-hydroxyglutarate (2-HG), instead of α-ketoglutarate	Enasidenib, Ivosidenib
Phosphofructokinase-2 (PFK-2)	Catalyzes the synthesis of fructose-2,6-bisphosphate from fructose-6-phosphate	PFK-015

## Data Availability

No new data were created or analyzed in this study. Data sharing is not applicable to this article.

## Abbreviations

TME: Tumor microenvironment
IDH: isocitrate dehydrogenase
NSCLC: non-small cell lung cancer
GLS1: glutaminase 1
LAT1: L-type amino acid transporter 1
OXPHOS: Oxidative phosphorylation
PPP: Pentose phosphate pathway
TCA: Tricarboxylic acid
LDHA: Lactate dehydrogenase A
2-HG: 2-Hydroxyglutarate
α-KG: Alpha-ketoglutarate
ACC: Acetyl-CoA carboxylase
MCT: Monocarboxylate transporter
CPT1: Carnitine palmitoyltransferase 1
HK2: Hexokinase 2
FH: Fumarate hydratase
PFK-2: Phosphofructokinase-2
mFFX: modified FOLFIRINOX
mTOR: Mammalian target of rapamycin
ccRCC: Clear cell renal cell carcinoma
BCAAs: Branched-chain amino acids
VHL: von Hippel-Lindau
