# Supplementary material for: Cancer Metabolism and Its Historical & Molecular Foundations: An Overview
Source: Drugs Drug Candidates. Author manuscript; Available in PMC 2026 Mar 20. (PMC13002032; doi:10.3390/ddc5010017)
Supplement: Suppl Info [file NIHMS2153107-supplement-Suppl_Info.pdf]

Table S1: Specific Candidates.

Candidate Drug	Target / Function	IC_{50} / EC_{50} (Reported Range)	Highest Clinical Stage (Oncology)	Key Side Effects / Limitations	Proposed Biomarker / Vulnerable Subgroup	Ref.
**V-9302**	ASCT2 (SLC1A5): Neutral amino acid transporter (glutamine uptake)	~10 µM (cellular uptake inhibition)	Preclinical	Potential disruption of systemic amino acid homeostasis; limited in vivo pharmacokinetic data.		[179]
**ND-646 (Firsocostat)**	ACC1/2: Acetyl-CoA carboxylase (fatty acid synthesis)	~3 nM (enzyme inhibition)	Phase II (NASH; limited oncology data)	Thrombocytopenia and GI intolerance were observed in metabolic disease trials.		[180,181]
**Etomoxir**	CPT1: Fatty acid oxidation	~5–10 µM	Discontinued (Phase III heart failure; not advanced in oncology)	Hepatotoxicity and cardiotoxicity due to on-target CPT1 inhibition in the heart and liver.		[182]
**Denifanstat (TVB-2640)**	FASN: Fatty acid synthase	~0.05 µM (52 nM)	Ph II/III astrocytoma (with bevacizumab); Ph II KRAS-mutant NSCLC	Ocular toxicity (dry eye, corneal changes), alopecia, and GI symptoms due to systemic lipid disruption.	FASN-overexpressing tumors (astrocytoma, breast cancer)	[125]
**BAY-876**	GLUT1: Glucose transporter	2–6 nM (GLUT1 selective)	Preclinical	Risk of hypoglycemia, anemia (GLUT1 in erythrocytes), GI toxicity; narrow therapeutic window expected.	GLUT1-dependent tumors	[183]
**WZB117**	GLUT family inhibitor (non-selective)	~280 nM (cellular glucose uptake)	Preclinical	Limited selectivity; potential systemic hypoglycemia; off-target effects.		[184]

Table S1: Specific Candidates.

**BPTES**	GLS1 (Kidney-type glutaminase): Glutaminolysis inhibition	~3–10 µM (biochemical); ~5–20 µM (cellular)	Preclinical	Poor aqueous solubility, low bioavailability, suboptimal pharmacokinetics, and limited in vivo efficacy despite strong mechanistic proof-of-concept.		[162,185]
**Telaglenastat (CB-839)**	GLS1: Glutaminase (glutaminolysis)	20–50 nM	CANTATA (Ph III RCC): negative in unselected; KEAPSAKE (Ph II NSCLC): terminated for futility	Fatigue, nausea, transaminitis; limited efficacy in late-stage trials.	KEAP1/NRF2-mutant NSCLC, ccRCC	[164,186]
**Devimistat (CPI-613)**	PDH, KGDH (Mitochondrial Metabolism)	~50-100 µM (cellular)	AVENGER 500 (Ph III PDAC): missed OS endpoint, met PFS endpoint	GI toxicity and fatigue	AML, possibly PDAC with specific metabolic profiles?	[134,135]
**IPN60090**	GLS inhibitor (brain-penetrant)	20–90 nM	Phase I	Early clinical development; safety profile under investigation.		[187]
**2-Deoxyglucose (2-DG)**	HK2 / Glycolysis inhibition	~1–2 mM (cellular systems)	Phase I/II (not advanced; limited efficacy)	Hyperglycemia, fatigue, QT prolongation, cardiac toxicity; poor therapeutic index.		[188]
**Oxamate**	LDHA: Lactate dehydrogenase A	~150 µM	Preclinical	Low potency; broad lactate suppression may impair normal tissues (muscle, brain).		[189]
**Nanvuranlat (JPH203)**	LAT1 (SLC7A5): Essential amino acid transport	0.06–0.14 µM	Phase III (biliary tract cancer, region-specific trials)	Nausea, diarrhea, fatigue, pruritus; on-target amino acid depletion.	LAT1-overexpressing biliary tract cancer	[190]

Table S1: Specific Candidates.

**IACS-010759**	Complex I (OXPHOS) inhibitor	~5–10 nM (cellular)	Phase I (AML, solid tumors)	Lactic acidosis, neuropathy, cardiac effects; high mitochondrial toxicity risk.		[191]
**Metformin**	Complex I (indirect inhibition; AMPK activation)	~1–10 mM (cellular IC50; plasma lower)	Phase II/III (multiple oncology trials)	GI intolerance common; lactic acidosis rare; weak potency at clinically achievable concentrations.		[192]
**AZD3965**	MCT1: Lactate transport inhibition	~1.6 nM	Phase I/II (DLBCL, solid tumors)	Retinal toxicity (MCT1 in photoreceptors), GI disturbances.	MCT1-expressing lymphomas, solid tumors	[193]
**Ivosidenib**	Mutant IDH1 inhibitor	~12 nM	FDA Approved (AML, Cholangiocarcinoma)	Differentiation syndrome, QT prolongation, fatigue, GI effects.	IDH1-mutant AML, cholangiocarcinoma	[147]
**Enasidenib**	Mutant IDH2 inhibitor	~100 nM	FDA Approved (AML)	Differentiation syndrome, hyperbilirubinemia, and nausea.	IDH2-mutant AML	[150]
**PFK-015**	PFKFB3: Glycolysis regulation	~20–200 nM	Preclinical	Anti-angiogenic effects; potential endothelial toxicity.		[194]
